# Evaluation of pediatric patients with drug allergy confirmed by in vivo diagnostic tests

**DOI:** 10.1111/pai.70334

**Published:** 2026-04-01

**Authors:** Kezban İpek Demir, Deniz Yılmaz, Funda Aytekin Güvenir, Tayfur Giniş, Candan İslamoğlu, Zeynep Şengül Emeksiz, Betül Karaatmaca, Müge Toyran, Ersoy Civelek, Emine Dibek Misirlioğlu

**Affiliations:** ^1^ Department of Pediatric Allergy/Immunology Ankara Bilkent City Hospital Ankara Turkey; ^2^ Department of Pediatric Allergy/Immunology University of Health Sciences Ankara Turkey

**Keywords:** drug provocation test, in vivo diagnostic tests, pediatric, skin tests

## Abstract

**Background and Objective:**

Adverse drug reactions (ADRs) are a serious public health problem. This study aimed to evaluate reaction characteristics and diagnostic test results in pediatric patients in whom suspected drug allergy was confirmed by in vivo diagnostic tests.

**Methods:**

Children who presented with suspected drug allergy between January 1, 2013 and January 1, 2023 in Ankara, Türkiye and underwent in vivo diagnostic testing were included in the study. Patients with positive diagnostic tests were compared with those whose tests resulted negative.

**Results:**

Over the 10‐year study period, 1818 patients presented with suspected drug allergy and were followed up for 1892 presumed drug‐associated hypersensitivity reactions. A total of 4532 in vivo diagnostic tests were performed with the 2082 suspect drugs implicated in these reactions. Drug allergy was confirmed by in vivo diagnostic tests in 225 patients (12.5%). A total of 242 (5.3%) tests resulted positive, with positivity rates of 1.3% for skin prick tests (SPTs), 2.7% for intradermal tests (IDTs), 19.4% for patch tests and 8.5% for drug provocation tests (DPTs). Among patients with positive in vivo diagnostic test results, the most common suspect drugs were antibiotics (53.9%), followed by non‐steroidal anti‐inflammatory drugs (NSAIDs; 31.5%) and antiepileptic drugs (7.4%). There was no significant difference between patients with positive and negative diagnostic test results in terms of sex (50.2% vs. 53.4%, *p* = .36). However, age at reaction was significantly higher among patients with positive diagnostic test results (92 vs. 49 months, *p* < .001). The prevalence of antibiotics (53.9% vs. 78.2%) was significantly higher and the rates of NSAIDs (31.5% vs. 15%) was significantly lower in the group with negative diagnostic test results (*p* < .001 for both). In both groups, the most common clinical manifestations were cutaneous symptoms (93.3% and 94.5%, *p* = .44). Respiratory symptoms and a history of anaphylaxis were more frequent in the group with positive test results.

**Conclusion:**

The use of standardized systematic approaches for the diagnosis and management of drug hypersensitivity reactions can potentially improve outcomes, and diagnostic tests should be performed in patients with no contraindications.

**Key message**

Use of standardized systematic approaches for the diagnosis and management of drug hypersensitivity reactions can potentially improve outcomes, and diagnostic tests should be performed in patients with no contraindications.

## 1 INTRODUCTION

Adverse drug reactions (ADRs) are a serious public health problem that affects physician prescribing and practice. The literature indicates that 0.4%–10% of pediatric hospitalizations can be attributed to ADRs, and 0.6%–16.8% of hospitalized children may experience an ADR.^1^ Cross‐sectional, survey‐based screening studies from different countries have reported drug allergy prevalences ranging from 2.8% to 7.8%.^2^, ^3^ However, when assessed with diagnostic tests, drug allergy can only be confirmed in 1%–5% of these patients.^2^, ^4^

In a large Turkish study conducted in a general pediatrics outpatient setting, although 7% of parents reported a history of drug allergy, only 4.2% of suspected cases were confirmed after comprehensive allergy evaluation and diagnostic testing, corresponding to a confirmed drug allergy prevalence of 0.05% in this population.^5^ These data confirm that ADRs pose a significant medical and public health problem among children.^6^ The purpose of evaluating patients presenting with suspected drug allergy is to verify whether the reaction is actually due to drug allergy and to recommend a safe alternative drug that can be used by the patient for similar indications if a definite or a high probability of drug allergy is diagnosed.^7^, ^8^ The results of diagnostic tests demonstrate that drug allergy is actually present in less than 40% of patients presenting with suspected drug allergy.^9^ This can lead to the unnecessary prescription of alternative drugs that are more expensive or less effective.^10^, ^11^ Therefore, in the absence of a contraindication, diagnostic testing is recommended for patients presenting with drug allergy. Tests used in the diagnosis of drug allergies include in vitro tests as well as in vivo tests such as skin tests and drug provocation tests (DPTs).^12^ DPTs remain the gold standard method for the definitive diagnosis of drug allergies.^13^, ^14^ A positive DPT result is diagnostic of drug allergy, while a negative result largely excludes the diagnosis.

In this study, we aimed to evaluate reaction characteristics, the diagnostic tests performed and their results in pediatric patients in whom suspected drug allergy was confirmed by in vivo diagnostic tests.

## 2 METHODS

The study included children who presented to Ankara Pediatric Hematology‐Oncology Training and Research Hospital between January 1, 2013 and September 1, 2019 and Ankara Bilkent City Hospital Pediatric Allergy and Immunology Department between September 1, 2019 and January 1, 2023 with suspected drug allergy and who underwent in vivo diagnostic tests (skin prick tests [SPT], intradermal test [IDT], patch test, or DPT). Patients' records were retrospectively reviewed to collect information regarding the demographic characteristics (age and sex) of the patients, the causative drug, and the characteristics (time and type) of the allergic reaction.

Drug reactions were classified by time of onset as occurring within 1 h or after more than 1 h following drug intake (within 24 h for nonsteroidal anti‐inflammatory drug [NSAID] reactions) and were categorized as immediate or delayed reactions.^14^, ^15^ Anaphylaxis was defined according to the criteria specified in the European Academy of Allergy and Clinical Immunology (EAACI) statement and the World Allergy Organization (WAO) 2020 anaphylaxis guideline; DRESS (drug reaction with eosinophilia and systemic symptoms) syndrome was defined according to the Registry of Severe Cutaneous Adverse Reactions (RegiSCAR) scoring system; and acute generalized exanthematous pustulosis (AGEP) was defined according to the EuroSCAR scoring system.^16^, ^17^ Other cutaneous manifestations (urticaria, angioedema, symmetric drug‐related intertriginous and flexural exanthema, fixed drug eruption, maculopapular exanthema [MPE], and Stevens‐Johnson syndrome/toxic epidermal necrolysis [SJS/TEN]) were defined according to the criteria set forth in the relevant EAACI position paper.^18^

In vivo diagnostic tests conducted with identified suspect drugs, confirmation rates of the diagnostic tests and the reactions that occurred during the diagnostic tests were evaluated. Patients with positive diagnostic test results (confirmed drug allergy) and those with negative results were compared. Exclusion criteria were as follows: not performing in vivo diagnostic procedures were not performed due to high‐risk clinical histories or contraindications, inability to complete the diagnostic work‐up.

### 2.1 Diagnostic tests

#### 2.1.1 Skin prick and intradermal tests

SPT and IDT were performed in patients with suspected IgE‐mediated reactions. The drugs were tested on the forearm skin using the prick method. An induration at least 3 mm larger in diameter than the negative control at 20 min after application was considered positive. IDT was performed if SPT resulted negative. Injectable forms were administered intradermally at the maximum non‐irritating dose and interpreted after 20 min. Histamine 10 mg/mL was used as the positive control and 0.9% NaCl as the negative control. Reactions were considered positive when the size of the initial wheal increased by 3 mm or more in diameter.^19^ For penicillins, SPT was performed using a penicillin test kit (major determinant poly‐L‐lysine [PPL]), minor determinant mixture (MDM) and penicillin G (10,000 IU/mL); injectable forms were used for other suspected drugs.^19^ For non‐β‐lactam drugs, injectable formulations were tested according to ENDA/EAACI‐recommended non‐irritating concentrations where available.^19^ For drugs without established validated non‐irritating concentrations, test concentrations were selected based on published studies defining non‐irritating doses in control populations.^20^, ^21^

#### 2.1.2 Patch tests

Patch tests were performed for delayed mild cutaneous ADRs (MPE, contact dermatitis and fixed drug eruption), and severe cutaneous drug reactions (AGEP, DRESS and TEN/SJS). Patch tests were performed at least 6 weeks after the drug reaction and at least 6 months after the drug reaction in patients presenting with severe drug reactions. Powder vial or tablet forms of suspect drugs were preferred, and concentrations of 5%, 10% and 30% were obtained by mixing with white petroleum jelly. The patches were applied to the upper back, between the shoulder blades. Reactions were evaluated after 48 and 72 h. The presence of erythema, indurations, and vesicles was considered a positive test result.

SPT, IDT and patch testing were performed using doses recommended in EAACI guidelines.^19^

#### 2.1.3 Drug provocation tests

In patients with negative SPT and IDT results, DPT was performed at least 4–6 weeks after the reaction. No provocation test was performed on patients with a history of severe cutaneous drug reaction. Direct drug provocation testing was applied in patients with non‐immediate, mild cutaneous reactions (including maculopapular exanthema and delayed urticaria), provided there was no history of severe cutaneous adverse reactions, systemic symptoms or anaphylaxis. Direct DPT was performed without prior skin testing in accordance with recent guideline recommendations and published pediatric cohort studies.^22^, ^23^ The ENDA guidelines were used to determine the indications, contraindications, and application of DPT.^24^ The age‐ and weight‐adjusted daily dose of the suspect drug was administered orally in 2–5 incremental doses given at 30‐min intervals. In case of any objective clinical findings (e.g., urticaria, rash, angioedema, hypotension, persistent vomiting, cough and wheezing), DPT was discontinued and considered positive. Patients were monitored in the clinic for at least 2 h after administration of the last dose.

### 2.2 Statistical analyses

Statistical analyses were performed using the SPSS v.22 statistical software package for Windows (IBM Corp., Armonk, NY). Numbers and percentages were reported for categorical variables. Continuous data were not normally distributed and were expressed as median and interquartile range (IQR; 25th–75th percentile). Chi‐square test was used to compare categorical data; the Mann–Whitney *U* test was used for comparisons of continuous variables that were not normally distributed. A *p*‐value of <0.05 was considered significant.

## 3 RESULTS

**FIGURE 1:**
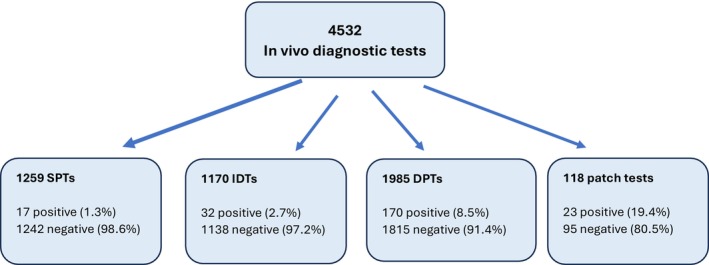
Distribution of the diagnostic tests.DPT, drug provocation test; IDT, intradermal test; SPT, skin prick test.

Over the 10‐year study period, 1818 patients presented with suspected drug allergy and were followed up for 1892 presumed drug‐associated hypersensitivity reactions. A total of 4532 in vivo diagnostic tests were performed with the 2082 suspect drugs implicated in these reactions. These tests included 1259 SPTs (27.7%), 1170 IDTs (25.8%), 118 patch tests (2.6%) and 1985 DPTs (43.7%). A total of 242 (5.3%) tests resulted positive, with positivity rates of 1.3% for SPT (*n* = 17), 2.7% for IDT (*n* = 32), 19.4% for patch tests (*n* = 23) and 8.5% for DPT (*n* = 170). Higher confirmation rates with DPT support its role as the definitive diagnostic standard for pediatric drug allergy. The remaining 4290 in vivo diagnostic tests resulted negative, with negativity rates of 98.6% for SPT (*n* = 1242), 97.2% for IDT (*n* = 1138), 80.5% for patch test (*n* = 95) and 91.4% for DPT (*n* = 1815) (Figure 1). Among confirmed β‐lactam reactions (*n* = 113), diagnoses were established by SPT in 6 (5.3%), IDT in 23 (20.3%), DPT in 80 (70.7%) and patch testing in four cases (3.5%). For non‐β‐lactam antibiotics (*n* = 10), confirmation was obtained by SPT in 2 (20%), IDT in 1 (10%), DPT in 6 (60%) and patch test in 1 case (10%). In the NSAID group (*n* = 72), 70 cases (97.2%) were confirmed by DPT and 2 (2.7%) by IDT. All confirmed antiepileptic reactions (*n* = 17) were diagnosed exclusively by patch testing. The relatively higher patch test positivity rate observed in our study compared with the literature may be attributed to our center being a tertiary referral center for drug allergy.

Of the 225 patients with positive diagnostic test results, 50.2% (*n* = 113) were male, the median age at reaction was 92 months (IQR 36–140 months), and the median age at diagnosis was 96 months (IQR 50–150 months). The median time from reaction to diagnostic testing was 4 months (IQR 2–11 months). Of the 1593 patients with negative diagnostic test results, 53.4% (*n* = 851) were male, the median ages at reaction and diagnostic testing were 49 months (IQR 18–96 months) and 68 months (IQR 32–115 months), respectively, for a median interval from reaction to diagnostic testing of 4 months (IQR 2–12 months). There was no significant difference between patients with positive and negative diagnostic test results in terms of sex (*p* = .36). Although age at reaction and age at diagnostic testing were significantly higher among patients with positive diagnostic test results (*p* < .001 for both), there was no significant difference between the two groups in terms of the time from reaction to diagnostic testing (*p* = .19) (Table 1). This age‐related difference is clinically relevant, as it suggests that older children are more likely to have true drug hypersensitivity, whereas reactions occurring at younger ages may more frequently represent transient or pseudoallergic reactions, often in the context of viral infections.

**TABLE 1:** Demographic characteristics (*N* = 1818).

	Patients with positive diagnostic tests	Patients with negative diagnostic tests	*p* ^a^
	*n* = 225 (12.3%)	*n* = 1593 (87.6%)	
Sex (male), *n* (%)	113 (50.2)	851 (53.4)	0.36
Age at reaction (months), median (IQR)	92 (36–140)	49 (18–96)	<0.001
Age at diagnostic testing (months), median (IQR)	96 (50–150)	68 (32–115)	<0.001
Time from reaction to diagnostic testing (months), median (IQR)	4 (2–11)	4 (2–12)	0.19

^a^ *p* Values of statistical difference between groups using either Mann–Whitney *U* test or Pearson's chi‐square test depending on the characteristics of the data.

Among patients with positive in vivo diagnostic test results, the most common suspect drugs were antibiotics (*n* = 123, 53.9%; β‐lactam antibiotics accounting for 91.9%, *n* = 113), followed by NSAIDs (*n* = 72, 31.5%) and antiepileptic drugs (*n* = 17, 7.4%). This distribution is clinically consistent, as antibiotics represent the most frequently prescribed drug group in childhood, followed by NSAIDs; therefore, these agents also constitute the most commonly confirmed culprit drugs in pediatric drug hypersensitivity cohorts. Among patients with negative diagnostic test results, this distribution was 78.2% antibiotics (*n* = 1451, β‐lactams accounting for 90.6%, *n* = 1315), 15% NSAIDs (*n* = 279) and 0.9% antiepileptic drugs (*n* = 16). The prevalence of antibiotics was significantly higher and the rates of NSAIDs and antiepileptic drugs were significantly lower in the group with negative diagnostic test results (Table 2).

**TABLE 2:** Comparison of suspected drugs used in diagnostic tests with positive and negative results (*n* = 2082).

	Positive diagnostic tests (*n* = 228)		Negative diagnostic tests (*n* = 1854)		*p* ^a^
	*n*	%	*n*	%	
Antibiotics β‐lactam Non‐β‐lactam	123 113 10	53.9	1451 1315 135	(78.2)	<0.001
NSAID Ibuprofen Paracetamol	72 40 18	31.5	279 140 125	15	<0.001
Antiepileptic Carbamazepine Valproic acid	17 10 4	7.4	16 5 2	0.9	<0.001
Other	16	(7.01)	108	5.8	

^a^ *p* Values of statistical difference between groups using Pearson's chi‐square test.

In terms of reaction characteristics, immediate reactions were significantly more prevalent in the positive group (47.1% vs. 17.7%), while delayed reactions were more common in the negative group (52.8% vs. 82.2%) (*p* < .001 for both). When grouped by diagnostic test, the prevalence of immediate reactions was 82.3% among children with positive SPT results, 84.3% among those with positive IDT results, and 46.4% among those with positive DPT results. In children with negative test results, this distribution was 46.2% for SPT, 45.6% for IDT, and 18.4% for DPT (*p* = .003, *p* < .001 and *p* < .001, respectively). All patients who underwent patch tests were those with delayed reactions.

The group with positive results also had a significantly higher prevalence of intravenous drug intake (10.5% vs. 4.3%, *p* < .001), suggesting an increased risk of true drug hypersensitivity, potentially related to faster systemic exposure and higher bioavailability associated with parenteral routes. In both groups, the most common clinical manifestations were cutaneous symptoms (93.3% and 94.5%, *p* = .44), followed by respiratory symptoms. However, respiratory symptoms were more frequent in the group with positive test results (14.5% vs. 2.8%, *p* < .001). A history of anaphylaxis was also significantly more common among patients with positive diagnostic test results (13.2% vs. 2.5%, *p* < .001) (Table 3). Reaction characteristics according to drug groups, including antibiotics, NSAIDs and antiepileptic drugs (*n* = 1795, 94.8%), are summarized in Table 3B. Other drugs (e.g., sedatives, anesthetics, proton pump inhibitors, enzymes and biological agents) represented only a minority (*n* = 97, 5.1%).

**TABLE 3:** (A) Reaction characteristics (*n* = 1892). (B) Reaction characteristics depending on drug groups (*n* = 1795)^b^.

(A)					
	Reactions with positive diagnostic test results (*n* = 227)		Reactions with negative diagnostic tests results (*n* = 1665)		*p* ^a^
	*n*	%	*n*	%	
Reaction characteristics					
Cutaneous	212	93.3	1575	94.5	0.44
Urticaria	98	43.1	710	42.6	0.1
Angioedema	78	34.3	278	16.6	<0.001
Maculopapular rash	50	2.2	648	38.9	<0.001
Respiratory	33	14.5	48	2.8	<0.001
Gastrointestinal	8	3.5	25	1.5	0.05
Neurologic	6	2.6	21	1.2	0.12
Cardiovascular	–	–	2	0.1	1
Severe drug reaction					
Anaphylaxis	30	13.2	42	2.5	<0.001
DRESS	3	1.3	11	0.6	0.23
SJS	4	1.7	5	0.3	0.016
AGEP	2	0.8	1	0.06	0.04
Time from last dose to reaction					
Immediate reactions (<1 h)	107	47.1	296	17.7	<0.001
Non‐immediate reactions (>1 h)	120	52.8	1369	82.2	<0.001
Route of administration					
Oral	184	81	1477	88.7	0.002
Intramuscular	19	8.4	115	6.9	0.49
Intravenous	24	10.5	73	4.3	<0.001

(B)									
	Antibiotics (*n* = 1465)			NSAID (*n* = 297)			Antiepileptic (*n* = 33)		
	Positive diagnostic test results (*n* = 123)	Negative diagnostic tests results (*n* = 1342)	*p* ^a^	Positive diagnostic test results (*n* = 72)	Negative diagnostic tests results (*n* = 225)	*p* ^a^	Positive diagnostic test results (*n* = 17)	Negative diagnostic tests results (*n* = 16)	*p* ^a^
	*n* (%)	*n* (%)		*n* (%)	*n* (%)		*n* (%)	*n* (%)	
Reaction characteristics									
Cutaneous	117 (95.1)	1276 (95)	0.98	67 (93)	220 (97.7)	0.05	17 (100)	16 (100)	–
Respiratory	19 (15.4)	30 (2.2)	<0.001	10 (13.8)	7 (3.1)	<0.001	–	–	–
Gastrointestinal	4 (3.2)	23 (1.7)	0.22	3 (4.1)	–	0.014	–	–	–
Neurologic	4 (3.2)	19 (1.4)	0.12	1 (1.3)	–	0.24	–	–	–
Cardiovascular	–	1 (0.07)	1	–	–	–	–	–	–
Time from last dose to reaction									
Immediate reactions	48 (39)	206 (15.3)	<0.001	48 (66.6)	63 (28)	<0.001	–	–	–
Non‐immediate reactions	75 (60.9)	1136 (84.6)	<0.001	24 (33.3)	162 (72)	<0.001	17 (100)	16 (100)	–
Route of administration									
Oral	91 (73.9)	1196 (89.1)	<0.001	72 (100)	225 (100)	–	17 (100)	15 (93.7)	0.48
Intramuscular	17 (13.8)	104 (7.7)	0.019	–	–	–	–	–	–
Intravenous	15 (12.1)	42 (3.1)	<0.001	–	–	–	–	1 (6.2)	0.48

^a^ *p* values of statistical difference between groups using Pearson's chi‐square test.

^b^ Reaction characteristics according to drug groups (antibiotics, NSAIDs and antiepileptics) are presented. Other drugs (e.g. sedatives, anesthetics, proton pump inhibitors, enzymes and biological agents) accounted for a minority (*n* = 97, 5.1%).

## 4 DISCUSSION

In this study, 1818 patients presented to the study centers with 1892 suspected ADRs, resulting in a total of 4532 in vivo diagnostic tests performed with 2082 identified suspect drugs. We confirmed drug hypersensitivity in only 5.3% of all in vivo diagnostic tests performed, with an overall confirmation rate of 12.5% among tested patients. These findings are consistent with previous pediatric cohorts demonstrating that although a history of suspected drug allergy is frequent, true drug allergy confirmed by diagnostic tests remains relatively uncommon, ranging between 5% and 25% depending on the population studied and the diagnostic protocols applied (Ponvert et al.; Caubet et al.; Arıkoğlu et al.).^25^, ^26^, ^27^

The in vivo diagnostic tests used included SPTs, IDTs, patch tests and DPTs. The sensitivity of skin tests is generally higher in immediate reactions than in delayed reactions. For this reason, SPTs are recommended as an initial diagnostic test, especially in cases requiring rapid diagnosis, because they are simple, fast, low cost and low risk. Although IDTs are a more sensitive method, this test carries the risk of causing irritative, false positive reactions and systemic reactions at high concentrations.^19^, ^28^, ^29^ In our study, 242 of the in vivo diagnostic tests resulted positive, confirming drug allergy. Of these, 17 (7%) were SPTs and 32 (13.2%) were IDTs following negative SPT. Similarly, in a separate cohort of 606 patients evaluated for suspected penicillin allergy, a total of 274 skin tests were performed, of which 14 (5.1%) yielded positive results.^30^

A total of 170 positive results were obtained through drug provocation, yielding a positivity rate of 8.5%. Consistent with our results, Vezir et al. evaluated the diagnostic performance and safety of DPT in a single‐center pediatric allergy clinic and reported that while SPTs were negative in all cases, and IDTs were positive in only 4% of patients, DPTs were positive in 6.8% of provocations performed with culprit drugs. These findings reinforce that clinical history alone is insufficient to establish a diagnosis of drug allergy and emphasize the critical role of comprehensive diagnostic evaluation, particularly DPT, in confirming or excluding drug hypersensitivity in children.^23^ The markedly higher confirmation rates observed with DPT, compared to other in vivo diagnostic modalities, reinforce its status as the gold standard for diagnosing drug hypersensitivity in children. Our findings align with the 2022 AAAAI/ACAAI Drug Allergy Practice Parameter, which emphasizes drug provocation testing as the cornerstone of diagnosis in pediatric drug hypersensitivity, and supports direct provocation without prior skin testing in selected low‐risk non‐immediate cutaneous reactions.^15^

Patch tests and/or delayed reading of the IDT can be performed for delayed drug reactions.^31^, ^32^, ^33^ However, there are no standardized and validated test concentrations for many drug allergens. In addition, false negativity may occur because allergic reactions often develop in response to an active metabolite, not the main drug. For this reason, DPTs are needed to confirm the diagnosis, but patch tests can be helpful in diagnosis, especially in severe drug reactions which are a contraindication for DPT. In our study, 23 patch tests resulted positive, corresponding to 9.5% of all positive tests and 19.5% of all patch tests performed. Although positivity rates vary widely in pediatric cohorts internationally (0.9%–89%), our findings appear relatively high, which may reflect the referral‐center profile of our institution and the higher proportion of patients evaluated for severe cutaneous adverse reactions.^34^, ^35^ Taken together, while patch tests alone are insufficient for definitive diagnosis, they remain clinically valuable in severe delayed reactions where DPT is contraindicated.

When stratified by drug group, most confirmed β‐lactam reactions were diagnosed by drug provocation (70.7%), with intradermal (20.3%), skin prick (5.3%) and patch testing (3.5%) contributing minimally. For non‐β‐lactam antibiotics, drug provocation accounted for 60% of cases. In the NSAID group, nearly all diagnoses (97.2%) were established by provocation, consistent with Arıkoğlu et al., who reported similar findings (27/31, 87.1% by provocation, 4/31, 12.9% by skin testing).^27^ For antiepileptics, all confirmed cases were identified exclusively by patch testing, in line with Atanasković‐Marković et al., who observed positive results in 89.4% of children with confirmed hypersensitivity.^35^ The negative predictive value (NPV) of all SPTs, early/late reading of the IDT and patch tests for the definitive diagnosis of drug allergy was found to be around 80%–90%.^36^, ^37^, ^38^ There are few standardized skin and in vitro tests for the diagnosis and management of drug hypersensitivity reactions; provocation tests are the gold standard method for diagnosing drug allergy.^24^ Therefore, drug allergy cannot be completely ruled out based on negative skin test results, and provocation tests should be performed unless contraindicated.

When the demographic characteristics of the patients with positive diagnostic test results and those with negative test results were compared, there was no significant difference between the two groups in terms of sex distribution. Although female sex in adults has been associated with an increased risk of drug hypersensitivity reactions, especially perioperative reactions, this sex difference has not been observed in children.^39^ In contrast, both the age at reaction and the age at diagnostic testing were significantly higher among patients with confirmed allergy. This age‐related gradient is clinically meaningful. Older children may have increased true drug hypersensitivity, whereas reactions in younger children more commonly reflect transient viral‐associated exanthems or pseudoallergic responses. In an international multicenter study, Rashed et al. demonstrated that the incidence of ADRs in hospitalized patients was significantly higher in children >11 years old compared with younger age groups.^40^ Similarly, Rubio et al. reported that a substantial proportion of suspected reactions in younger children were subsequently attributed to infectious causes rather than true hypersensitivity.^41^ Taken together, these findings indicate that increasing age is associated with a higher probability of confirmed drug hypersensitivity, whereas suspected reactions in younger children should be interpreted carefully because viral exanthems frequently mimic drug eruptions.

Several studies have reported that antimicrobial drugs are the most common cause of ADRs, followed by NSAIDs.^3^, ^42^, ^43^ In accordance with the literature, antibiotics were the most common suspect drugs both in patients with positive and with negative in vivo diagnostic test results (53.9% and 78.2%, respectively), followed by NSAIDs (31.5% and 15%, respectively) and antiepileptic drugs (7.4% and 0.9%, respectively). In both groups, over 90% of the antibiotics were β‐lactams. However, the overall proportion of antibiotics was significantly higher in the group with negative test results, while the proportion of NSAIDs and antiepileptic drugs was lower. Bacterial and viral exanthems, which are important in the differential diagnosis of drug allergy, are much more common in young children.^44^, ^45^ As patients often receive antibiotic treatment during these infections, the skin lesions that occur can be interpreted as drug‐related reactions. This explains why β‐lactam antibiotics appeared more frequently as a suspect drug in the group with negative diagnostic test results. The proportion of NSAID‐related reactions among patients with confirmed drug allergy (31.5%) represents a clinically relevant finding in our cohort. Previous Turkish pediatric studies have shown that NSAID hypersensitivity is frequently overestimated based on clinical history alone, with confirmation rates ranging from approximately 20%–38% after standardized diagnostic evaluation (Topal et al., 20.3%; Arıkoğlu et al., 31.1%; Simsek et al., 37.5%; Cavkaytar et al., 27%).^22^, ^46^, ^47^, ^48^ NSAID hypersensitivity may occur via two main mechanisms: cross‐intolerance, most commonly related to cyclooxygenase‐1 (COX‐1) inhibition, and selective reactions mediated by immunologic pathways.^49^ In children, NSAID hypersensitivity may often present as a cross‐intolerance phenotype. Given that therapeutic alternatives are more limited in pediatric practice, unnecessary NSAID allergy labeling may lead to clinically significant treatment restrictions. Therefore, confirmation with validated diagnostic tools, drug provocation testing, is essential to ensure accurate diagnosis and avoid inappropriate long‐term drug avoidance.

Non‐immediate cutaneous reactions were markedly more frequent among patients with negative diagnostic outcomes, suggesting that a substantial proportion of these may not represent true drug hypersensitivity. In pediatric practice, viral exanthems are particularly common and may be misinterpreted as drug‐related eruptions, especially when antibiotics are prescribed during intercurrent infections. Supporting this, Zambonino et al. reported that more than 90% of children evaluated for suspected β‐lactam allergy were ultimately found to be tolerant after complete diagnostic work‐up.^50^ Similarly, Rubio et al. showed that many delayed cutaneous eruptions in children are attributable to infectious causes rather than immunologically mediated drug hypersensitivity.^41^

Among positive test results, immediate reactions constituted 82.3% of positive SPT, 84.3% of positive IDT and 46.4% of positive DPT outcomes, confirming that immediate‐type hypersensitivity is more readily detected by current diagnostic tools. Our findings are consistent with those of previous large pediatric cohorts. Ponvert et al. reported that 86% of immediate‐type hypersensitivities to β‐lactams were identified by skin tests, whereas 68.4% of non‐immediate‐type hypersensitivities were diagnosed only by challenge testing. Similarly, Kitsos et al. demonstrated in their pediatric cohort that while SPT and IDT showed high specificity, their sensitivity remained low; in particular, a substantial proportion of delayed cutaneous reactions could only be confirmed through drug provocation testing.^51^ In line with these findings, Kulhas Celik et al. reported that among 191 pediatric patients with a history of immediate reactions to penicillin, diagnostic tests confirmed penicillin allergy in 36 patients (18.8%), of whom 23 (63.8%) were diagnosed by positive skin tests and 13 (26.2%) by drug provocation tests.^52^

In addition, cutaneous symptoms were the most common clinical manifestation in both groups (93.3% and 94.5%) and the second most common were respiratory symptoms, which were significantly more common in the group that had positive diagnostic test results (14.5% vs. 2.8%). Patients with drug allergy confirmed by diagnostic tests were also significantly more likely to have a history of anaphylaxis (13.2% vs. 2.5%). In children, cutaneous symptoms (especially MPE) are the most frequently reported reactions, followed by gastrointestinal symptoms.^2^, ^4^, ^10^, ^53^ Symptoms can also include isolated respiratory reactions (mostly limited to NSAIDs) or, more commonly, respiratory symptoms as part of an anaphylactic reaction.^54^ Test sensitivity is generally better in immediate reactions than delayed reactions.^55^, ^56^ This explains the higher frequency of anaphylaxis and immediate reactions in the group with positive diagnostic test results. In addition, as infectious exanthems typically manifest as MPE, they may be mistaken for delayed drug reactions. This may explain the higher prevalence of delayed reactions in the group with negative results.

The drugs implicated in the reactions were most commonly administered orally in both groups, although the prevalence of oral drugs was higher in the group with negative diagnostic test results (81% vs. 88.7%). Intravenously administered drugs accounted for a significantly larger proportion of confirmed culprit drugs in the group with positive results (10.5% vs. 4.3%). The association between intravenous drug administration and confirmed drug allergy observed in our study is clinically relevant. Parenteral administration is known to result in rapid systemic exposure and higher peak drug concentrations, which may facilitate immune recognition and increase the likelihood of hypersensitivity reactions compared with oral administration.^14^ Thus, intravenous administration is a known risk factor for drug hypersensitivity reactions.

As the study involved a retrospective review of 10‐year data, some data were not available. This represents a limitation of the study. One of the main strengths of this study is that it was conducted at a single reference center with significant experience in drug allergy. In addition, the use of standardized diagnostic methods increases the reliability of the results.

## 5 CONCLUSION

In this 10‐year, single‐center pediatric cohort, diagnostic evaluation confirms drug hypersensitivity in only a minority of children with suspected reactions. β‐lactam antibiotics and NSAIDs remain the most frequently implicated drug groups. Drug provocation testing provides the highest diagnostic yield and represents the cornerstone for confirming or excluding drug hypersensitivity when not contraindicated. In vivo tests are safe and clinically useful in children when selected according to the underlying hypersensitivity mechanism. Skin prick and intradermal tests contribute mainly in immediate reactions, whereas patch testing has selective value in severe delayed cutaneous reactions where provocation is unsafe. These findings highlight the importance of mechanism‐based, standardized diagnostic pathways in pediatric practice to prevent inappropriate long‐term drug allergy labeling and to maintain access to first‐line therapeutic options.

## AUTHOR CONTRIBUTIONS

**Kezban İpek Demir:** Conceptualization; investigation; resources; software. **Deniz Yılmaz:** Validation; visualization. **Funda Aytekin Güvenir:** Validation; visualization. **Tayfur Giniş:** Formal analysis; project administration. **Candan İslamoğlu:** Writing – review and editing. **Zeynep Şengül Emeksiz:** Formal analysis; project administration; funding acquisition. **Betül Karaatmaca:** Writing – original draft; data curation. **Müge Toyran:** Supervision. **Ersoy Civelek:** Formal analysis; validation. **Emine Dibek Misirlioğlu:** Methodology; writing – review and editing; resources; supervision.

## FUNDING INFORMATION

There are no sources of funding to declare.

## CONFLICT OF INTEREST STATEMENT

The authors have no conflicts of interest to declare.

## ETHICS STATEMENT

Consent was obtained from families (and patients older than 9 years) for study participation and all diagnostic tests. The study was approved by the Ankara Bilkent City Hospital Ethics Committee (decision number: E2‐23‐3595).
